# Mentalization deficit in bipolar patients during an acute depressive and manic episode: association with cognitive functions

**DOI:** 10.1186/s40345-017-0107-3

**Published:** 2017-12-06

**Authors:** Anna Bodnar, Janusz K. Rybakowski

**Affiliations:** Department of Adult Psychiatry, Poznan University of Medical Sciences, ul.Szpitalna 27/33, 60-572 Poznan, Poland

**Keywords:** Bipolar disorder, Mentalization, Theory of mind, Cognitive mentalization, Affective mentalization, Bipolar depression, Mania, Cognitive functions

## Abstract

**Background:**

A number of studies in bipolar patients have shown a deficit in mentalization (theory of mind), one of the main aspects of social cognition. The aim of current study was to assess both cognitive and affective mentalization in well-defined groups of depressed and manic bipolar patients, compared to healthy control subjects, using a battery of tests measuring mentalization processes. The second aim was to investigate a possible relationship between cognitive and affective mentalization and cognitive functions in bipolar patients during a depressive and manic episode.

**Methods:**

The study involved 25 bipolar disorder type I patients (10 male, 15 female) during a depressive episode (mean 24 ± 2 points in the 17-item Hamilton Depression Rating Scale) and 25 patients (10 male, 15 female) during a manic episode (mean 27 ± 4 points in the Young Mania Rating Scale). The control group consisted of 25 healthy subjects (10 male, 15 female) without psychiatric disorders. To measure mentalization, a revised version of the Reading the Mind in the Eyes (R-MET), the Strange Stories (SS), the Faux Pas Recognition (FPR), and the Moving Shapes Paradigm (MSP) tests were used. Assessment of cognitive functioning was made using the Digit Span, Trail Making, and Wisconsin Card Sorting Tests.

**Results:**

In bipolar patients significant deficits in both cognitive and affective mentalization were demonstrated during both acute depressive and manic episodes. The impairment in FPR in manic patients was more severe than that in the depressive ones. On the other hand, in MSP, manic patients showed significantly increased intentionality for non-mentalization animations, compared with depressive patients and for “cause and effect” animations compared with control subjects. A significant relationship was found between the decrease in cognitive and affective mentalization and deficits of cognitive functions during both the depressive and manic episodes.

**Conclusions:**

The results obtained confirm the deficits of mentalization in bipolar I patients, during both acute depressive and manic episodes. We found that in such patients mentalization deficits significantly correlated with cognitive dysfunctions more so during depressive episodes.

## Background

The ability to mentalize (mentalization, theory of mind—ToM) about other people’s mental and emotional states forms one of the main aspects of social cognition. This term was introduced by Premack and Woodruff (1978), on the basis of their study of chimpanzees, as an ability to explain the activities and behaviors observed in other individuals by referring to their mental status. The past two decades of mentalization studies in humans have lead to a proposal that mentalization contains both cognitive and affective elements (Shamay-Tsoory et al. 2007). Cognitive mentalization is an inference about the cognitive mental state, mainly beliefs, of other persons. Affective mentalization is an inference about the emotional state of other persons. Distinguishing cognitive and affective mentalization has also been proposed on the basis of studies which demonstrated different neural correlates of these two domains. Hynes et al. (2006), in a functional magnetic resonance study, showed that the medial orbitofrontal lobe, defined as Brodmann’s areas 11 and 25, was preferentially involved in affective mentalization. This corresponded with Shamay-Tsoory et al. (2006) study showing an impairment of affective mentalizations in subjects having lesions in the ventromedial prefrontal cortex. On the other hand, Kalbe et al. (2010) using a repetitive transcranial magnetic stimulation (rTMS) approach, indicated the right dorsolateral prefrontal cortex (DLPFC) as the brain structure mostly connected with cognitive mentalization.

Previous research has suggested that impaired mentalization (ToM) forms the core handicap in autism subjects (Happé 1994). Also, two decades ago, a deficit in mentalization was demonstrated in schizophrenia (Frith and Corcoran 1996) and, in subsequent studies, it has been confirmed as an essential feature of this illness (Green et al. 2015). The first study on mentalization in bipolar disorder (BD) was performed by Kerr et al. (2003). This involved 20 patients during a manic episode, 15 in a depressive episode, 13 in remission, and 15 control subjects and used a method of six short stories. They found deficits in mentalization during manic and depressive episodes where the mean intensity of mania was 13 points, according to the Bech Mania Rating Scale, and 16 points for depression, according to the Beck Depression Inventory. In subsequent research, using tests of higher levels of complexity, the authors demonstrated that such deficits in BD also occur during remission periods (Inoue et al. 2004; Bora et al. 2005). Montag et al. (2010) demonstrated that bipolar patients scored significantly lower than control subjects for the cognitive aspect of ToM but not for the affective one. In recent meta-analyses of this topic, a deficit in ToM in bipolar patients in both acute episodes and during the euthymic state has been evidenced (Samamé et al. 2015; Bora et al. 2016). However, lately, Haag et al. (2016) did not show differences in visual theory of mind abilities between euthymic bipolar patients and healthy controls.

In some mentalization research studies, a concomitant assessment of cognitive functions has been performed. In a few, a possible relationship between mentalization and cognitive functions has been suggested. In the study of Bora et al. (2005), BD patients displayed an impairment in many cognitive tasks, including those related to sustained attention. Lahera et al. (2008) showed impaired sustained attention and executive functions in such patients. In the study of Ioannidi et al. (2015) BD patients had poorer performances in verbal memory and visuospatial working memory during both the acute and euthymic phases of the illness. Impaired immediate memory and executive functions were only noted during episodes of illness. Olley et al. (2005) showed an impaired performance on the Stroop task in remitted BD patients and a correlation between ToM and the CANTAB Stocking of Cambridge task. However, Wiener et al. (2011) while finding significant impairments on the subtests of CANTAB battery in BD patients, did not find a relationship between these tests and mentalization results. Recently, Van Rheenen et al. (2014) showed a significant correlation between tests measuring social cognition and neurocognition but not between neurocognition and emotional regulation.

It is thus conceivable that mentalization abnormalities could be connected with manic and/or depressive symptoms of BD. Therefore, in this study, an attempt was made to assess both cognitive and affective mentalization in well-defined groups of manic and depressed bipolar patients, compared to healthy control subjects, using a battery of tests measuring mentalization processes. We also hypothesized that, during an acute manic and depressive episode such abnormalities can be connected with cognitive functions. Therefore, the second aim of the study was to investigate a possible relationship between cognitive and affective mentalization and cognitive functions in these groups of bipolar patients and healthy subjects.

## Methods

### Participants

The study was performed on 50 patients with bipolar mood disorder type I (20 male, 30 female) aged 19–65 years, with a duration of illness of 11 ± 10 years. They were hospitalized in the inpatient clinic of the Department of Adult Psychiatry, Poznan University of Medical Sciences, on account of an acute manic or depressive episode of BD. A consensus diagnosis of BD was made for each patient by at least two psychiatrists according to DSM-IV criteria (SCID) (First et al. 1996). Exclusion criteria covered any other psychiatric co-morbidity or serious medical condition.

Twenty-five patients (10 male, 15 female), aged 41 ± 14 years, were studied during a depressive episode, and 25 patients (10 male, 15 female) aged 35 ± 14 years during an acute manic episode. The criterion for inclusion in the study for depressive patients was an intensity of depression, as assessed by the 17-item Hamilton Depression Rating Scale (HDRS) (Hamilton 1980)—of ≥ 18 points and for manic patients an intensity of mania, as assessed by the Young Mania Rating Scale (YMRS) (Young et al. 1978)—of ≥ 20 points.

On the day of study, the patients received pharmacological treatment: antidepressant and/or mood-stabilizing drugs in depression and antipsychotic and/or mood-stabilizing drugs in mania. The number of patients (in parentheses), taking individual drugs was as follows: lithium (23), olanzapine (20), valproate (20), quetiapine (16), venlafaxine (10), haloperidol (8), paroxetine (8), lamotrigine (5), aripiprazole (4), risperidone (4), carbamazepine (4), topiramate (2), bupropion (1), escitalopram (1), mirtazapine (1).

A comparison group consisted of ten healthy men and 15 healthy women, aged 36 ± 11 years who had volunteered to be studied in response to an internet advertisement. They did not report any psychiatric disturbance in themselves or in any 1st degree members of their family.

The number of years of education was 13 ± 3 years in depressed patients, in manic patients 15 ± 3 years, and in control subjects 16 ± 2 years.

### Assessment of mentalization

A revised version of the Reading the Mind in the Eyes Test, the Strange Stories Test, the Faux Pas Recognition Test, and the Moving Shapes Paradigm were used for the measurement of mentalization. Previously, these scales had been translated into Polish by three independent translators, then, the best version was selected and back-translation performed. The reliability of the tests was assessed by the Cronbach’s alpha factor.

#### The Reading Mind in Eyes Test (R-MET)

The revised version of the R-MET consists of 36 photos presenting the eyes and their adjacent areas. All the pictures show exactly the same region of the face—from the middle of the nose to the area above the brows. The subject being studied is asked to choose one of the four terms which best describes what the person on the picture is thinking or feeling. This test assesses the ability to identify both cognitive and affective mental states because, for inferring what the person on the picture is thinking or feeling it includes, among others, such terms as suspicious (cognitive component) and fearful (affective component) (Baron-Cohen et al. (2001)).

#### Strange Stories (SS)

This tool was introduced by Happé (1994) and it assesses the ability to understand a lie, a white lie, a figurative expression, irony, or persuasion. After presentation of each story, the subject is asked to answer a question connected with its content. A correct answer requires an inference about the mental state of the character in the story. In this study, five stories for assessing cognitive mentalization, as well as five control stories requiring inferences about physical causality are used.

#### The Faux Pas Recognition Test (FPR)

This test was introduced by Stone et al. (1998). By definition, a faux pas occurs when someone says something without thinking that the person who hears it may not wish to hear it or be offended or hurt by it. The test used in this study consists of ten stories arranged in random order—five stories with social condition mistakes and five control stories. The subject being studied is asked to read the story and answer a few questions connected with it. In the understanding and recognition of the Faux Pas, both cognitive and affective mentalizations are involved.

#### Moving Shapes Paradigm (MSP)

This is a collection of 12 computer animations designed by Abell et al. (2000). Each lasts 34–35 s and presents two moving triangles: one large red and one small blue. In this study three groups of four animations each, were used: (1) random animations: triangles moving about aimlessly without interactions; (2) cause-and-effect animations: the triangles are interacting: following, fighting, dancing, etc.; (3) mentalization animations: the triangles are mutually reacting to each other’s mental states (e.g., 1 triangle is trying to seduce, to ridicule, or to scare the other). The subject studied is asked to view the animations which are presented in a pseudo-random sequence and answer a question: What happened during the animation? Two criteria are assessed: appropriateness of the attribution and the correctness of the intentionality of the story (random, cause-and-effect, mentalization).

### Assessment of cognitive functioning

Assessment of cognitive functioning was made using The Digit Span, Trail Making and Wisconsin Card Sorting Tests.

#### Digit Span Test (DST)

This test is one of the subtests of the Wechsler Adult Intelligence Scale-Revised. The first part consists of remembering and presenting a series of digits in the correct order, and measures short-term auditory memory. The second part consists of remembering and presenting a series of digits in the reverse of the order presented, and measures verbal working memory.

#### Trail Making Test (TMT)

The Polish version of the Trail Making Test (TMT) is an element of the Halstead–Reitan Test battery (Reitan 1958). Part A of the test consists of connecting, by a continuous line, the points between 1 and 25 as quickly as possible and measures psychomotor speed and visual–motor coordination. Part B consists of connecting, by a continuous line as quickly as possible, the digits alternatively with consecutive letters i.e., 1 – *A* – 2 – *B* − 3 – *C*, etc., and reflects the ability to shift strategy and assess executive function and visuospatial working memory. The results, separately for *A* and *B*, are presented as the duration of performance (in seconds), and the number of errors.

#### Wisconsin Card Sorting Test (WCST)

The Wisconsin Card Sorting Test (WCST) assesses the executive functions, controlling cognitive processes and the strategy of solving problems. It is also a tool for the assessment of working memory, abstract thinking, and “set shifting,” i.e., the ability to display flexibility in the face of changing schedules of reinforcement. The subject is asked to match the 64 cards, according to color, shape, and number of elements. He/she is not told how to match. However, the subject is informed whether a particular match is right or wrong. After ten correct matches, the criterion is changed without informing the participant. The test is terminated when six categories are correctly arranged or all the cards are used. Percentages of errors, of perseverative responses, of conceptual responses, and the number of completed categories are calculated. A computer version of the WCST (Heaton 1981) was used in this study.

### Experimental procedure

The experimental procedure consisted of two parts, over 2 days, each lasting about 1 h, performed by one of the authors (AB). On the first day, mentalization tests were performed. On the second day, empathy and cognitive functions were assessed. At the time of study, clinical assessment by the HDRS or YMRS scale in the patient was performed by a psychiatrist who was taking care of the patient in the ward.

### Statistics

The one-way analysis of variance (ANOVA) with Tukey’s post hoc test was performed. The belongingness to the three groups: depressed, manic, and control subjects made the independent variable. The dependent variables included selected components of mentalization and cognitive functions as well as clinical factors. The r-Pearson Test was used to determine any correlation between the variables. The calculations were done using the IBM SPSS 21.0. The level of statistical significance was determined at *p* < 0.05.

## Results

As calculated by the analysis of variance, the three groups studied did not show differences due to age: *F*(2,72) = 1.37; *p* = 0.26. They did, however, differ as to years of education: *F*(2,72) = 7.96; *p* = 0.001. The control group had higher levels of education compared to the depressed group (*p* < 0.01) but not compared to the manic group: (*p* = 0.32). The patients with depression had marginally lower levels of education compared to the manic ones (*p* = 0.06).

On the day of study, the mean intensity of depression in the depressive patients, as measured with HDRS, was 24 ± 2 points and the mean intensity of mania in the manic patients, as measured by YMRS, was 27 ± 4 points.

For mentalization measures, assessed in the whole sample, the internal consistency coefficients were good, with a Cronbach’s alpha for the Reading the Mind in the Eyes: total 0.72, affective 0.52, cognitive 0.63; for the Strange Stories: total 0.83, control stories 0.70, mentalization stories 0.77, for the Faux Pas Recognition: total 0.91, affective 0.68, cognitive 0.86; and for the Moving Shapes Paradigm Tests: appropriateness 0.70, intentionality 0.52.

The results of the Reading the Mind in the Eyes, Strange Stories, and Faux Pas Recognition Test are presented in Table 1.

**Table 1:** The results of mentalization assessed by the Reading the Mind in the Eyes (R-MET), Strange Stories (SS), and Faux Pas Recognition (FPR) tests during depression (*A*), mania (*B*) and in healthy control subjects (*C*)

Mentalization	Group						Analysis of variance			Post hoc test		
	Depression		Mania		Controls							
	Mean	SD	Mean	SD	Mean	SD	*F*(2,72)	*p*	Eta^2^	*A* − *B*	*A* − *C*	*B* − *C*
R-MET-total	21.36	5.12	23.32	3.94	28.12	3.41	16.99	< 0.001	0.32	0.235	< 0.001	< 0.001
R-MET-cognitive	11.16	3.08	12.04	2.68	15.00	1.83	15.17	< 0.001	0.30	0.454	< 0.001	< 0.001
R-MET-affective	10.20	2.87	11.28	2.34	13.12	2.55	8.08	≤ 0.001	0.18	0.311	< 0.001	0.038
SS-total	11.64	3.57	9.96	3.74	17.24	2.05	35.28	< 0.001	0.50	0.161	< 0.001	< 0.001
SS-mentalization stories	6.56	2.02	5.48	2.20	8.96	1.17	23.09	< 0.001	0.39	0.106	< 0.001	< 0.001
SS-control stories	5.08	2.23	4.48	2.33	8.24	1.45	24.43	< 0.001	0.40	0.555	< 0.001	< 0.001
FPR-total	64.52	7.63	59.00	9.82	75.08	4.01	29.3	< 0.001	0.45	0.031	< 0.001	< 0.001
FPR*-*cognitive	20.16	3.91	17.52	4.89	26.32	2.61	33.22	< 0.001	0.48	0.051	< 0.001	< 0.001
FPR-affective	7.64	1.63	6.36	1.80	9.20	1.04	21.74	< 0.001	0.38	0.011	0.002	< 0.001

There were significant differences between groups in the Reading the Mind in the Eyes Test. Post hoc comparisons revealed a deficit in mentalization, both cognitive and affective, measured by this test, in both the depressive and manic patients, compared to healthy subjects. This was also the case for the Strange Stories Test, where both depressive and manic patients obtained significantly worse results than the control subjects, for both mentalization and control stories.

Likewise, there were significant differences between groups in the Faux Pas Recognition Test. Post hoc comparisons revealed a significant reduction in ability for such recognition in both depressive and manic patients, compared to healthy subjects. Furthermore, manic patients scored worse in this test, compared to the depressive ones. Such an impairment applied to both cognitive and affective mentalization in both groups of bipolar patients, and the manic patients had greater deficits in affective mentalization, compared to depressed ones.

All the differences above remained valid after corrections for multiple testing.

The results of the Moving Shapes Paradigm are shown in Table 2.

**Table 2:** The results of mentalization assessed by the Moving Shapes Paradigm during depression (*A*), mania (*B*) and in healthy control subjects (*C*)

Moving Shapes Paradigm	Group						Analysis of variance			Post hoc test		
	Depression		Mania		Controls							
	Mean	SD	Mean	SD	Mean	SD	*F*(2.72)	*p*	Eta^2^	*A* − *B*	*A* − *C*	*B* − *C*
Mentalization animations	2.00	1.12	2.24	1.16	3.16	0.75	8.89	< 0.001	0.20	0.688	< 0.001	0.006
Cause-and-effect animations	3.00	1.12	2.48	1.05	3.48	0.71	6.58	0.002	0.15	0.150	0.197	0.002
Random animations	2.08	1.29	1.12	1.01	3.16	0.99	21.34	< 0.001	0.37	0.008	0.003	< 0.001
Affective mentalization	2.96	1.57	2.88	1.45	4.24	1.59	6.16	0.003	0.15	0.982	0.012	0.007
Appropriateness total	11.24	4.08	9.64	2.84	17.00	3.77	28.75	< 0.001	0.44	0.266	< 0.001	< 0.001
Appropriateness mentalization	3.04	1.81	3.28	1.43	5.68	1.49	21.14	< 0.001	0.37	0.855	< 0.001	< 0.001
Appropriateness cause-and-effect	4.76	2.20	4.20	1.38	5.48	1.50	3.42	0.038	0.09	0.492	0.313	0.029
Appropriateness random	3.52	2.10	2.16	1.75	6.20	2.06	27.01	< 0.001	0.43	0.046	< 0.001	< 0.001
Intentionality total	26.80	6.27	32.84	5.24	28.28	3.62	9.30	< 0.001	0.21	< 0.001	0.571	0.007
Intentionality mentalization	12.72	3.16	13.96	2.61	16.28	2.05	11.69	< 0.001	0.25	0.228	< 0.001	0.008
Intentionality cause-and-effect	9.76	2.40	11.72	2.32	9.64	1.87	6.99	0.002	0.16	0.007	0.980	0.004
Intentionality random	4.72	3.59	7.56	3.68	2.36	2.66	15.20	< 0.001	0.30	0.010	0.039	< 0.001

Significant differences between groups were also observed for mentalization abilities as measured by the Moving Shapes Paradigm Test. Post hoc comparisons revealed a deficit in both cognitive and affective mentalization, measured by this test, in both depressive and manic patients, compared to healthy subjects. These differences remained valid after corrections for multiple testing.

For the mentalization and random animations both depressed and manic patients scored lower than the control subjects, while, in “cause and effect” animations, this was only the case for manic patients. Both groups were impaired in affective mentalization and on appropriateness for the total, mentalization and random animations. These differences remained valid after corrections for multiple testing.

However, for appropriateness of “cause and effect” animations, this was only observed in manic patients (not valid after multiple testing). For intentionality, both groups showed an impaired score in mentalization animation (valid after multiple testing) and increased scores in random animations (valid after multiple testing only for mania). For intentionality, total and “cause and effect,” manic patients scored higher than those depressed (valid after multiple testing only for “cause and effect”) and, in “cause and effect” animations higher than the control subjects (not valid after multiple testing).

For each of the four mentalization tests the differences between groups did not change when the results were controlled for the years of education.

The results of all the cognitive tests (Digit Span Test, TMT *A*, TMT *B*, WCST: percentage of errors, percentage of perseverative responses, completed categories, percentage of conceptual responses) were lower in both manic and depressive patients compared to healthy subjects while no difference between depressive and manic patients was found. To assess any possible relationship between mentalization measures and cognitive function tests, a correlation analysis of the results obtained in depressive and manic episodes was performed and is presented in Tables 3 and 4.

**Table 3:** Relationship between mentalization assessed by the Reading the Mind in the Eyes (R-MET), Strange Stories (SS), Faux Pas Recognition (FPR), and Moving Shapes Paradigm (MSP) tests and the results of cognitive function tests: a correlation analysis in a depressive episode

	Digit Span Test	TMT A	TMT B	WCST percentage of errors	WCST percentage of perseverative responses	WCST completed categories	WCST percentage of conceptual responses
Mentalization R-MET-total	0.330	− 0.452*	− 0.341	− 0.622**	− 0.469*	0.575**	0.533**
Mentalization FPR-total	0.214	− 0.438*	− 0.507**	− 0.685**	− 0.430*	0.445*	0.704**
Cognitive mentalization R-MET	0.378	− 0.446*	− 0.277	− 0.556**	− 0.555**	0.473*	0.413*
Cognitive mentalization SS	0.302	− 0.257	− 0.065	− 0.532**	− 0.616**	0.504*	0.505*
Cognitive mentalization FPR	0.025	− 0.328	− 0.328	− 0.553**	− 0.272	0.330	0.618**
Cognitive mentalization MSP	0.164	− 0.420*	− 0.579**	− 0.561**	− 0.352	0.522**	0.631**
Affective mentalization R-MET	0.183	− 0.329	− 0.310	− 0.513**	− 0.241	0.519**	0.508**
Affective mentalization FPR	− 0.046	− 0.104	− 0.291	− 0.436*	− 0.220	0.228	0.552**
Affective mentalization MSP	0.216	− 0.165	− 0.305	− 0.342	− 0.301	0.372	0.282

* *p* < 0.05; ** *p* < 0.01

**Table 4:** Relationship between mentalization assessed by the Reading the Mind in the Eyes (R-MET), Strange Stories (SS), Faux Pas Recognition (FPR), and Moving Shapes Paradigm (MSP) tests and the results of cognitive function tests: a correlation analysis in a manic episode

	Digit Span Test	TMT *A*	TMT *B*	WCST percentage of errors	WCST percentage of perseverative responses	WCST completed categories	WCST percentage of conceptual responses
Mentalization R-MET-total	0.240	− 0.218	− 0.322	− 0.392	− 0.297	0.257	0.424*
Mentalization FPR-total	− 0.291	0.297	0.246	− 0.435*	− 0.257	0.420*	0.424*
Cognitive mentalization R-MET	0.292	− 0.184	− 0.458*	− 0.240	− 0.069	0.164	0.272
Cognitive mentalization SS	− 0.131	0.001	− 0.335	− 0.277	− 0.107	0.264	0.160
Cognitive mentalization FPR	0.112	− 0.157	− 0.095	− 0.430*	− 0.194	0.423*	0.442*
Cognitive mentalization MSP	0.225	− 0.518**	0.015	0.102	− 0.174	− 0.080	− 0.094
Affective mentalization R-MET	0.070	− 0.156	− 0.017	− 0.387	− 0.422*	0.246	0.403*
Affective mentalization FPR	− 0.132	0.019	− 0.048	− 0.546**	− 0.240	0.567**	0.583**
Affective mentalization MSP	− 0.037	− 0.488*	− 0.026	− 0.212	− 0.133	0.158	0.143

* *p* < 0.05; ** *p* < 0.01

An analysis of a relationship between mentalization and cognitive function tests during depression did not find any correlations between mentalization and the Digit Span Test. On the other hand, 32 correlations were obtained between the four mentalization tests and the results of the TMT and WCST. A higher level of general mentalization was associated with better cognitive planning and efficiency of thinking, as well as with a fewer number of errors and perseverative responses in WCST. A better performance on the Faux Pas Recognition Test was connected with psychomotor speed, visual perception, visual–spatial coordination, and working memory. A higher level of cognitive mentalization was associated with better cognitive planning and efficiency of thinking (except for FPR), with a fewer number of errors and perseverative responses in WCST (R-MET and SS) and with psychomotor speed, visual perception, and visual–spatial coordination, and working memory (MSP). A higher level of affective mentalization was associated with a fewer number of errors and a higher percentage of conceptual responses in WCST (R-MET, FPR).

As in depression, the analysis of a relationship between mentalization and cognitive function tests during mania did not find any correlations between mentalization and the Digit Span Test. Fifteen correlations were obtained between the four mentalization tests and the results of the TMT and WCST. A higher level of general mentalization was associated with better cognitive planning. Better performance on the Faux Pas Recognition Test was connected with efficiency of thinking and fewer errors on WCST. A higher level of cognitive mentalization assessed by MSP was associated with better psychomotor speed and visual–spatial coordination and, when assessed by the R-MET test, with better attention and visual–spatial working memory. A higher level of affective mentalization was associated with better cognitive planning, a fewer number of perseverative responses (R-MET), better efficacy of thinking (FPR), and better psychomotor speed and visual–motor coordination.

In both tables no correlation coefficient was 0.64 or higher which would be necessary to withstand correction for multiple testing.

## Discussion

The first finding of our study is the demonstration of deficits in mentalization (theory of mind) in bipolar patients, during both acute manic and depressive episodes. In our study, this was evidenced by the use of a number of tests for measuring mentalization These included the Reading the Mind in the Eyes, the Strange Stories, the Faux Pas Recognition Tests, and the Moving Shapes Paradigm Tests. The results obtained indicate an impairment of both cognitive and affective mentalization in the groups of bipolar patients studied. This corresponds to the results of recent meta-analyses (Samamé et al. 2015; Bora et al. 2016).

Further analysis of our mentalization results showed that there were some differences in this respect between depressive and manic patients. In the Faux Pas Recognition Test the impairments in manic patients were more severe than in the depressive ones in both the total as well as cognitive and affective scores. On the other hand, in the Moving Shapes Paradigm Test, manic patients showed significantly increased intentionality for non-mentalization animations, compared to depressive patients and, for “cause and effect” animations, compared to the control subjects. This may be connected with the fact that manic patients perceive social stimuli from strangers as more positive and directed at them and not as random behavior (Piff et al. 2012). Increased perception of intentionality in manic patients could also correspond with the increased affective empathy (overempathising) we reported in bipolar patients during a manic episode (Bodnar and Rybakowski 2017).

The second finding of our study is that, in bipolar patients during an acute episode, mentalization deficits significantly correlated with cognitive dysfunctions. Impaired neurocognitive functioning in BD, more marked during an acute episode, is a well-demonstrated phenomenon (Tsitsipa and Fountoulakis 2015). In the current study we found many correlations between all the four mentalization tests applied and the results of such neuropsychological tests as TMT and WCST which measure psychomotor speed, visual–motor coordination, visuospatial working memory, and executive functions. Such correlations were more numerous in depressive than in manic patients. This may correspond to other studies pointing to cognitive dysfunctions in BD, concomitant to mentalization disturbances (Bora et al. 2005; Lahera et al. 2008; Wiener et al. 2011; Ioannidi et al. 2015), as well as to a relationship between mentalization and cognitive functions reported in such patients (Olley et al. 2005; Van Rheenen et al. 2014).

Disturbances of mentalization, as well as their relationship with cognitive dysfunctions during acute episodes of BD, should be regarded in the context of brain abnormalities in this illness. The neural circuits underlying the pathophysiology of mood disorders mostly include the prefrontal cortex and anatomically related limbic structures (Price and Drevets 2012). According to Strakowski et al. (2006) the main role is played by the anterior limbic network comprising the ventral and orbital parts of the prefrontal cortex, connected reciprocally with the amygdala, parahippocampal gyrus, insula, and the anterior part of the cingulated gyrus. As mentioned previously, the ventral and orbital parts of the prefrontal cortex have been suggested for affective mentalization (Hynes et al. 2006; Shamay-Tsoory et al. 2006) while Kalbe et al. (2010) suggests that the brain structure mostly associated with cognitive mentalization is the right dorsolateral prefrontal cortex (DLPFC). However, a recent study of Yeh et al. (2015) did not find differences between groups of patients having DLPFC vs ventromedial prefrontal cortex damage to both cognitive and affective aspects of mentalization.

In our study we obtained significant correlations between the results of the four tests measuring mentalization studies, and such cognitive function tests as the TMT and WCST. A meta-analysis of structural neuroimaging studies showed that the performance on these tests is related to the volume of the prefrontal cortex, mainly the lateral and medial parts of it (Yuan and Raz 2014). Recent studies have also shown an association between the dorsomedial prefrontal cortex and TMT B performance (Miskin et al. 2016) and between DLPFC and WCST (Boschin et al. 2016). This may indicate that a DLPFC abnormality in bipolar patients plays a role for both mentalization deficits and cognitive disorders. Such an abnormality and the resulting disturbances are less intense in BD patients than in schizophrenia (Bora and Pantelis 2016) as was confirmed in our study on WCST performance (Rybakowski et al. 2006).

Although the discussion above mainly concerned the prefrontal cortex it should be indicated that other areas of the brain may be also important for mentalization processes. According to a meta-analysis of Molenberghs et al. (2016), besides the prefrontal cortex, the second most important brain structure for mentalization is the temporoparietal junction. However, there is no data on the involvement of the temporoparietal junction in such cognitive tests as TMT and WCST.

Based on the differences obtained between manic and depressive patients, and especially for an increased intentionality for random and “cause and effect” animations in manic patients, it can be speculated that these are connected with differences in functional brain activities in these episodes. An example of this can be the right amygdala–hippocampal connectivity, increased in mania and decreased in depression (Li et al. 2015).

## Conclusions

The limitation of our study is that the bipolar patients were actively treated during the study, receiving antidepressant and/or mood-normalizing drugs in depression and antipsychotic and/or mood-normalizing drugs in mania. Another limitation may be a difference in the mean education level between the bipolar depressive patients and the control group. On the other hand, the strength of our study is the use of four test for measuring mentalization and the well-designed groups of depressive and manic patients. Therefore, bearing these limitations in mind, we believe that our results strongly suggest that mentalization is significantly impaired during acute episodes of BD disorder although there may be some differences between depressive and manic episodes. Such impairments significantly correlate with the results of neuropsychological tests such as TMT and WCST.

## Abbreviations

BD: bipolar disorder
DLPFC: dorsolateral prefrontal cortex
DST: Digit Span Test
FPR: Faux Pas Recognition Test
HDRS: Hamilton Depression Rating Scale
MSP: Moving Shapes Paradigm
R-MET: Reading the Mind in the Eyes Test
rTMS: repetitive transcranial magnetic stimulation
SS: Strange Stories
ToM: theory of mind
WCST: Wisconsin Card Sorting Test
YMRS: Young Mania Rating Scale
